# Evaluation of [^18^F]Favipiravir in Rodents and Nonhuman Primates (NHP) with Positron Emission Tomography

**DOI:** 10.3390/ph16040524

**Published:** 2023-03-31

**Authors:** Jian Rong, Chunyu Zhao, Xiaotian Xia, Guocong Li, Achi Haider, Huiyi Wei, Jiahui Chen, Zhiwei Xiao, Yinlong Li, Xin Zhou, Hao Xu, Thomas L. Collier, Lu Wang, Steven H. Liang

**Affiliations:** 1 Department of Radiology and Imaging Sciences, Emory University, 1364 Clifton Rd, Atlanta, GA 30322, USA; jian.rong@emory.edu (J.R.); chunyu.zhao@emory.edu (C.Z.); ahmed.haider.wahauib@emory.edu (A.H.); jiahui.chen@emory.edu (J.C.); zwxiao2022@whu.edu.cn (Z.X.); yinlong.li@emory.edu (Y.L.); xin.zhou@emory.edu (X.Z.); leecollier@mac.com (T.L.C.); 2 Division of Nuclear Medicine and Molecular Imaging, Massachusetts General Hospital & Department of Radiology, Harvard Medical School, Boston, MA 02114, USA; xiaotian_xia@hust.edu.cn; 3 Center of Cyclotron and PET Radiopharmaceuticals, Department of Nuclear Medicine and PET/CT-MRI Center, The First Affiliated Hospital of Jinan University, Guangzhou 510630, China; ligc2019@foxmail.com (G.L.); hy.wei0914@foxmail.com (H.W.); txh@jnu.edu.cn (H.X.)

**Keywords:** favipiravir, COVID-19, SARS-CoV-2, PET, F-18

## Abstract

The COVID-19 pandemic has posed a significant challenge to global public health. In response, the search for specific antiviral drugs that can effectively treat the disease caused by the SARS-CoV-2 virus has become a priority. While significant progress has been made in this regard, much work remains to address this ongoing crisis effectively. Favipiravir is an antiviral drug initially developed for the treatment of influenza and has received approval for emergency use for COVID-19 in many countries. A better understanding of the biodistribution and pharmacokinetics of Favipiravir in vivo would facilitate the development and translation of clinical antiviral drugs for COVID-19. Herein, we report the evaluation of [^18^F]Favipiravir in naive mice, transgenic mice models of Alzheimer’s disease, and nonhuman primates (NHP) with positron emission tomography (PET). The [^18^F]Favipiravir was obtained in an overall decay-corrected radiochemical yield of 29% with a molar activity of 25 GBq/µmol at the end of synthesis (EOS). PET imaging in naive mice, transgenic mice models of Alzheimer’s disease, and nonhuman primates revealed a low initial brain uptake, followed by a slow washout of [^18^F]Favipiravir in vivo. The [^18^F]Favipiravir was eliminated by a combination of hepatobiliary and urinary excretion. The low brain uptake was probably attributed to the low lipophilicity and low passive permeability of the drug. We hope this proof-of-concept study will provide a unique feature to study antiviral drugs using their corresponding isotopologues by PET.

## 1. Introduction

With the outbreak of SARS-CoV-2 infection in 2019, the covid pandemic has caused a severe public health crisis. More than 600 million infected cases and 6 million death cases have been reported across the globe [1]. As no specific drug was approved as the treatment for COVID-19 at the beginning of the pandemic, the repurposing of antiviral drugs was considered a potential treatment for COVID-19. Favipiravir is an antiviral drug initially developed for the treatment of influenza [2,3]. As a purine nucleic acid analog, Favipiravir is a potent virus RNA-dependent RNA polymerase (RdRp) inhibitor, and Favipiravir has been considered in several clinical trials for the treatment of COVID-19 [4]. Favipiravir is a pyrazine carboxamide derivative that has been reported to exhibit activities against RNA viruses in vivo and in vitro [3]. Favipiravir is phosphoribosylated to its corresponding ribofuranosyl-5′-triphosphate, which inhibits the activity of RNA-dependent RNA polymerase (RdRp) as a substrate. Since various RNA viruses have the conserved catalytic domain in RdRp, this inhibitory mechanism enables Favipiravir to exhibit a broader spectrum of antiviral activities [5]. It is reported that Favipiravir is also active against influenza A/B/C, including oseltamivir-resistant variants [5,6]. Further, some preclinical research has indicated that Favipiravir may have efficacy against Ebola [7].

In February 2020, Favipiravir was one of the repurposed antivirals for SARS-CoV-2 infection treatment [8,9,10,11]. The results showed that patients who tested positive for COVID-19 and were treated with Favipiravir got a negative virus test after four days. In contrast, patients’ test turned negative about 11 days later without Favipiravir (placebo) treatment. In the same trial, lung conditions improved in about 91% of patients taking Favipiravir [12,13,14,15,16]. In March 2020, a pilot trial suggested that Favipiravir was effective in treating COVID-19, and clinical trials are underway in Japan (Phase III), [17] Italy (Phase III) [18], and the U.S. (Phase I&II) [19,20]. Although Remdesivir and Favipiravir both target RdRp, it is worth mentioning that, Favipiravir is a fluorinated antiviral drug, representing a unique opportunity for studying dosing and target engagement in vivo using its ^18^F-isotopologue by positron emission tomography (PET).

PET is a widely used noninvasive imaging technique in disease diagnosis and drug development [21]. Notably, PET has a high sensitivity, and radioactive probes in trace amounts (ng-μg level) are sufficient for high-quality images. PET imaging with [^18^F]Favipiravir would be a facile strategy that provides insight into the drug’s biodistribution and pharmacokinetics properties in vivo and contributes to the design of clinical studies.

**Scheme 1:**
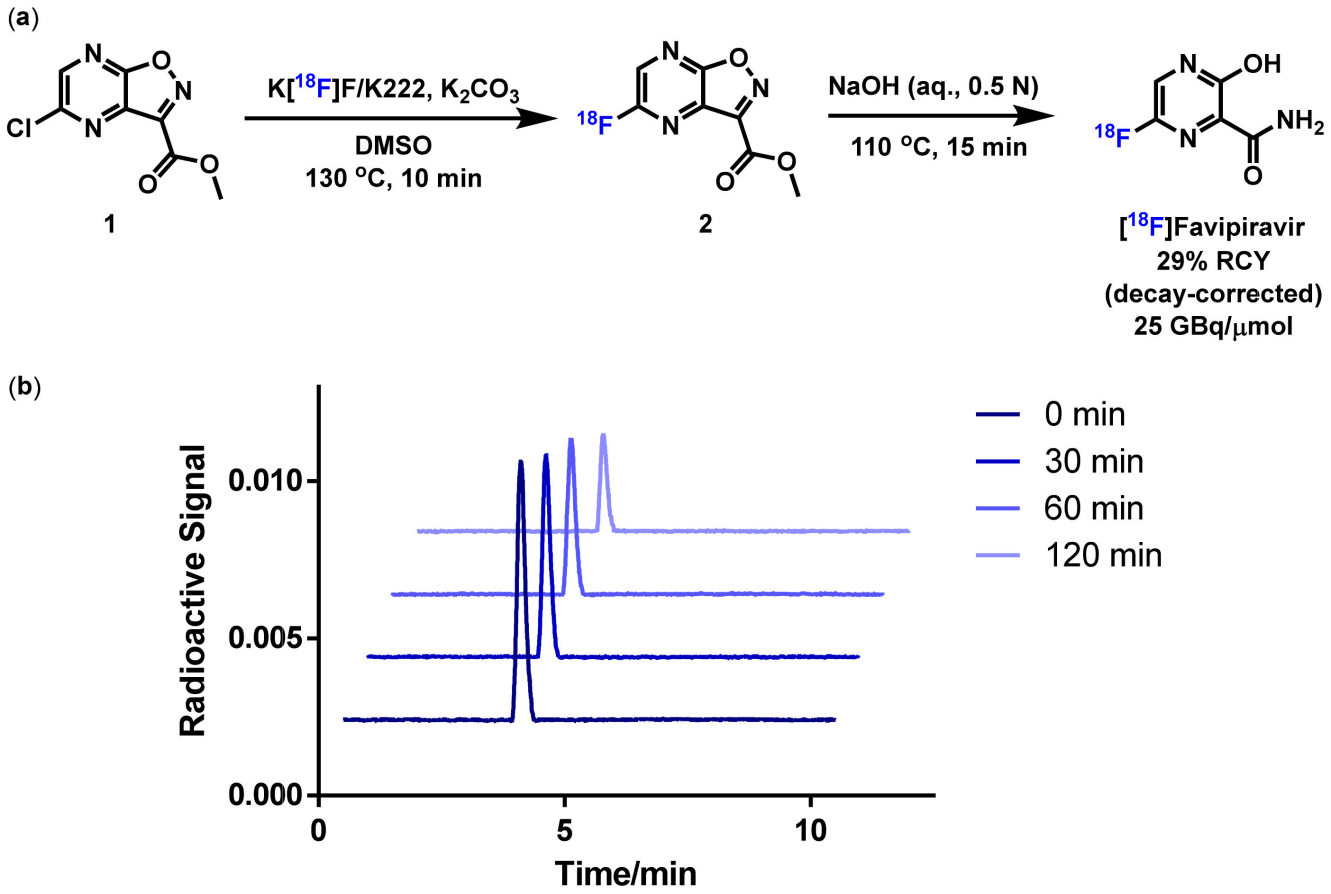
[^18^F]Favipiravir. (**a**) Synthesis of [^18^F]Favipiravir; (**b**) Stability of formulated [^18^F]Favipiravir analyzed using an HPLC radiochromatogram (a XBridge C18 column (5 µm, 4.6 mm × 100 mm) was used with an eluent of acetonitrile/H_2_O (*v*/*v* = 3/97, containing 0.01 M H_3_PO_4_) at 1 mL/min).

Although some preliminary positive outcomes have been made in COVID-19 patients with Favipiravir treatment, the study was limited to monitoring the drug level in plasma following treatment. We hypothesize that noninvasive imaging tools could provide the direct and real-time correlation between drug treatment and disease stages via whole-body distribution and target occupancy of Favipiravir in the organs of interest, such as the brain. PET can provide such information via targeted radioactive molecules (radiotracers). In this work, the tracer is [^18^F]Favipiravir; Scheme 1, which will be highly advantageous for monitoring [^18^F]Favipiravir exposure in different organs. A previous radiosynthesis and PET imaging study of [^18^F]Favipiravir on naïve mice [22] by Bocan et al. was reported, which encouraged us to further explore the potential of [^18^F]Favipiravir in disease models. We hypothesize that Favipiravir may exhibit different distributions in the central nervous systems (CNS) of rodents, nonhuman primates, and rodents under normal and neurodegenerative conditions, which can cause blood-brain barrier dysfunction/neuroinflammation.

Herein, we report the radiosynthesis and evaluation of [^18^F]Favipiravir in rodents and nonhuman primates (NHP) with PET to investigate the biodistribution and pharmacokinetic properties of Favipiravir. As a proof-of-concept, PET imaging studies with radiolabeled drugs would provide valuable insight for the design of clinical trials with new or repurposed antivirals for COVID-19.

## 2. Results

### 2.1. Radiochemistry

[^18^F]Favipiravir was prepared via nucleophilic aromatic substitution (S_N_Ar) reactions of the corresponding precursor, methyl-5-chloroisoxazolo [4,5-b] pyrazine-3-carboxylate, in the presence of K[^18^F]F/K_222_ and K_2_CO_3_ as the base in DMSO at 130 °C for 10 min, followed by hydrolysis with NaOH (0.5 N in water) at 110 °C for 15 min (Scheme 1) according to a previous report [22].

### 2.2. PET Imaging in Naive Mice with [^18^F]Favipiravir

**Figure 1:**
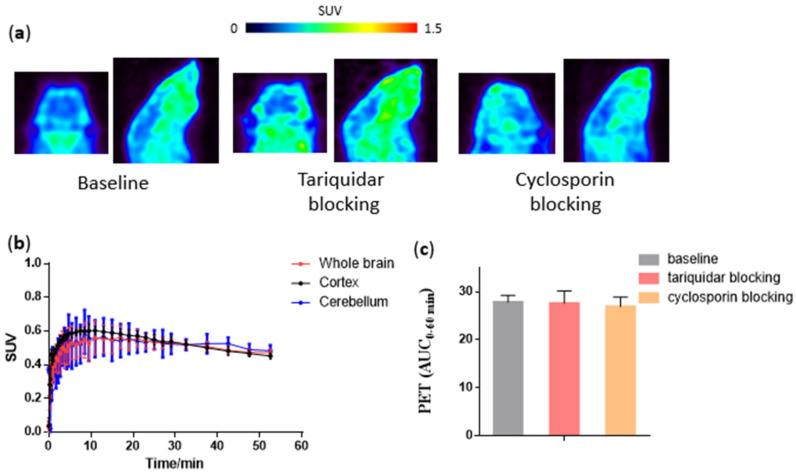
PET imaging of CD-1 mice under baseline and P-glycoprotein (P-gp) blocking conditions. (**a**) Representative summed PET images (0–60 min) of CD-1 mouse brains in the baseline and P-gp blocking studies; (**b**) TACs (0–60 min) of [^18^F]Favipiravir in CD-1 mouse brains under the baseline conditions (*n* = 4); (**c**) AUCs (0–60 min) of [^18^F]Favipiravir in CD-1 mouse brains in the baseline and P-gp blocking studies (*n* = 4 for baseline studies and *n* = 3 for blocking studies).

In dynamic PET imaging studies, CD-1 mice were imaged from 0 to 60 min following intravenous (i.v., *n* = 4) [^18^F]Favipiravir administration. The brain uptake of [^18^F]Favipiravir reached its maximum value of 0.6 SUV (standard uptake value) at around 10 min post-tracer administration and steadily declined to 0.45 SUV at 60 min post-tracer administration (Figure 1). To investigate the effect of P-glycoprotein (P-gp) efflux on brain uptake, we performed PET imaging of [^18^F]Favipiravir in mice pre-treated with Tariquidar or Cyclosporin, inhibitors of P-gp. In brief, CD-1 mice were treated with Tariquidar (10 mg/kg) [23] or Cyclosporin (25 mg/kg) [24] 15 min prior to [^18^F]Favipiravir administration. However, there was no significant difference in brain distribution of [^18^F]Favipiravir between the baseline and blocking studies with the inhibitors of P-gp (Figure 1).

### 2.3. PET Imaging in Transgenic AD and WT Mice with [^18^F]Favipiravir

**Figure 2:**
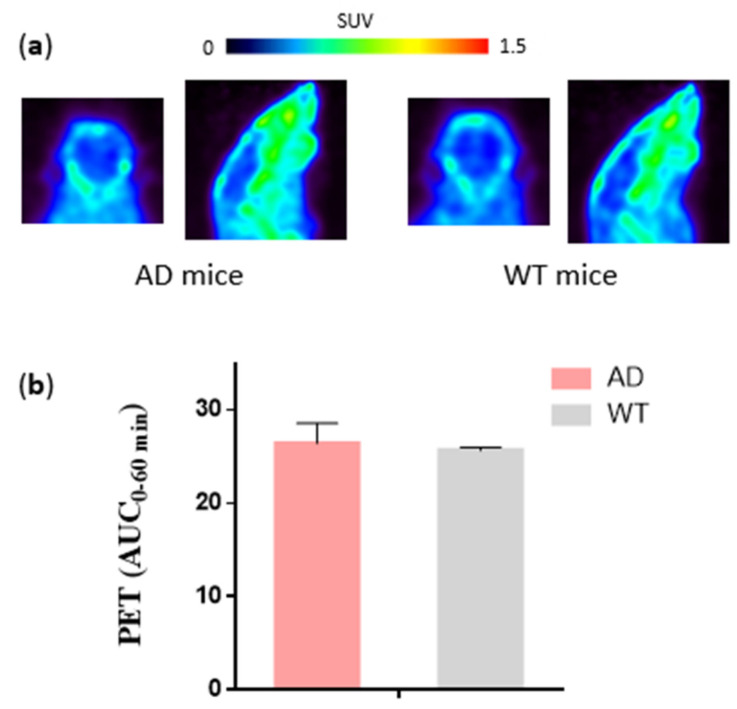
PET imaging of transgenic mice models of Alzheimer’s disease (AD) and age-matched wild-type (WT) controls with [^18^F]Favipiravir. (**a**) Representative summed PET images (0–60 min) of AD mouse brains and WT mouse brains with [^18^F]Favipiravir; (**b**) AUCs of [^18^F]Favipiravir in the brain of AD mice and WT mice (*n* = 3).

To investigate the brain penetration of [^18^F]Favipiravir between normal and neurodegenerative disease conditions, we performed PET imaging studies with [^18^F]Favipiravir in transgenic AD mice (5 × FAD; strain name: B6SJL-Tg(APPSwFlLon, PSEN1*M146L*L286V)6799Vas/Mmjax) and wild-type (WT) mice. The [^18^F]Favipiravir showed low brain uptake in both AD and WT mice (*n* = 3). No significant difference was found in the distribution of [^18^F]Favipiravir between AD and WT mice (Figure 2).

### 2.4. PET Imaging in NHP with [^18^F]Favipiravir

**Figure 3:**
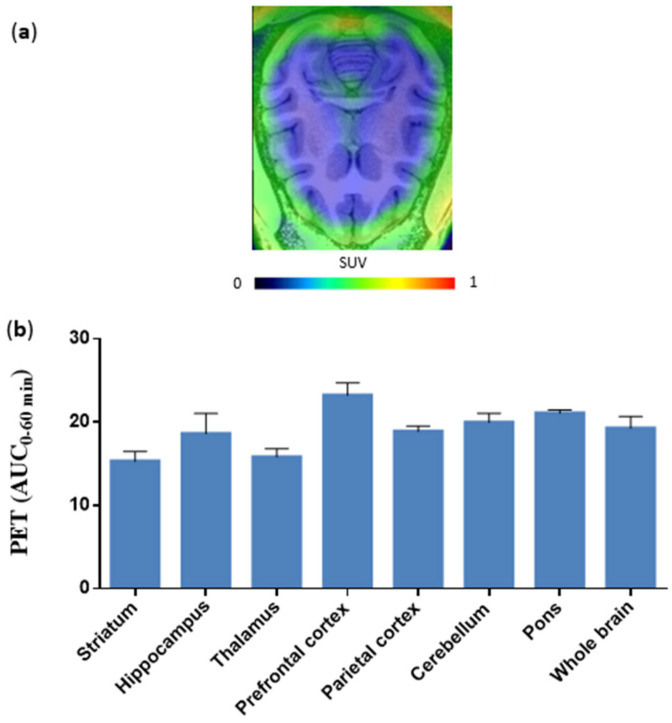
PET imaging of NHP brain with [^18^F]Favipiravir. (**a**) Representative summed PET images (0–60 min) of NHP brain with [^18^F]Favipiravir; (**b**) AUCs of [^18^F]Favipiravir in the NHP brain (*n* = 2).

The results of the evaluation of [^18^F]Favipiravir in the rhesus monkey PET study are shown in Figure 3. The [^18^F]Favipiravir displayed low brain penetration in the rhesus monkey brain. Heterogeneous distribution of [^18^F]Favipiravir was observed, and the highest radioactivity level was found in the prefrontal cortex, followed by the hippocampus, cerebellum, and pons. The lowest radioactivity uptake was observed in the striatum and thalamus. In addition, high radioactivity uptake was observed in the peripheral heart and lungs (Figure S3).

### 2.5. Ex Vivo Whole-Body Biodistribution in Mice with [^18^F]Favipiravir

**Figure 4:**
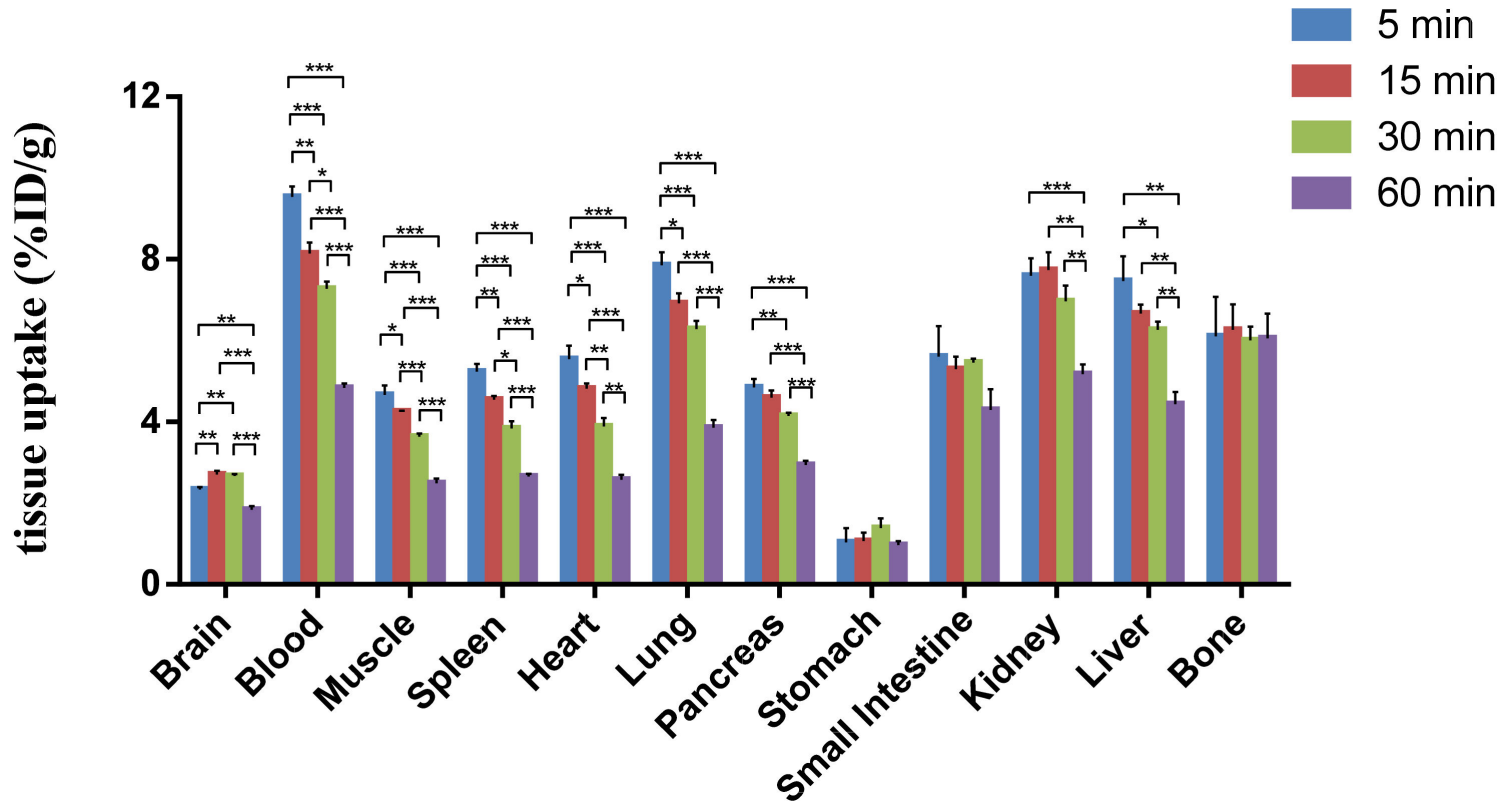
Whole-body ex vivo biodistribution study of [^18^F]Favipiravir in mice (*n* = 4). Statistical analysis was achieved using a student’s two-tailed *t*-test with asterisks indicating the statistical significance: * *p* < 0.05, ** *p* ≤ 0.01, and *** *p* ≤ 0.001.

To further investigate the distribution of [^18^F]Favipiravir in the whole body, we performed the ex vivo biodistribution experiment in CD-1 mice. Figure 4 and Table S1 show the uptake of [^18^F]Favipiravir in various organs of mice at 5, 15, 30, and 60 min post-tracer administration, respectively. At 5 min post-administration of [^18^F]Favipiravir, the radioactivity mainly accumulated in the blood, lung, kidney, and liver, with around or above 8% ID/g. After the initial high uptake in these organs, the radioactivity decreased over time. The remaining high radioactivity uptake in the small intestine and liver, at 60 min post-tracer administration, indicated a possible hepatobiliary and urinary excretion. From 5 min to 60 min post-tracer administration, no increase in bone uptake was observed over time.

### 2.6. Metabolic Analysis of [^18^F]Favipiravir in Mice

To investigate the in vivo stability of [^18^F]Favipiravir, we analyzed the radioactivity in the mouse brain and plasma. The parent contents of [^18^F]Favipiravir in the mouse brain and plasma were determined to be 41% and 89% at 30 min post-tracer administration, respectively (Figure S4).

## 3. Discussion

The radiosynthesis of [^18^F]Favipiravir involved an efficient transformation of nucleophilic aromatic substitution (S_N_Ar) reactions with the chloro precursor 1 with [^18^F]fluoride and hydrolysis with base. As a result, [^18^F]Favipiravir was obtained in an overall decay-corrected radiochemical yield of 29% with a molar activity of greater than 25 GBq/µmol at the end of synthesis (EOS). The [^18^F]Favipiravir had a radiochemical purity greater than 99%, and no obvious decomposition was detected up to 120 min after formulation with 5% EtOH in saline (Scheme 1).

In the PET imaging studies, as depicted in Figure 1, [^18^F]Favipiravir exhibited low yet steady brain uptake and relatively slow washout kinetics in CD-1 mice, which is consistent with a previous PET imaging study on naïve mice [22]. Administration of a P-gp inhibitor, Tariquidar (10 mg/kg) or Cyclosporin (25 mg/kg) prior to tracer injection, failed to increase the brain uptake of [^18^F]Favipiravir substantially. Hence, the interaction of [^18^F]Favipiravir with P-gp is possibly not the main reason for the relatively low SUV in the brain, and the [^18^F]Favipiravir is not the substrate of P-gp. The low brain uptake was probably attributed to the low passive permeability of Favipiravir, particularly resulting from its three H-bonding donors. In addition, Favipiravir has a low LogP of 0.72, which is in the suboptimal range for a CNS drug. The low lipophilicity of Favipiravir may in part contribute to its low brain uptake.

In the whole-body ex vivo biodistribution study in CD-1 mice, the high radioactivity accumulated in the blood, lung, kidney, and liver initially and then cleared over time in these organs. The remaining high radioactivity uptakes in the small intestine and liver, at 60 min post-tracer administration, indicated a possible combination of hepatobiliary and urinary excretion. The bone uptake of [^18^F]Favipiravir remained around 6%ID/g without increase over time, which suggested no significant in vivo de-radiofluorination for [^18^F]Favipiravir consistent with our PET observation. In the metabolic analysis of [^18^F]Favipiravir in CD-1 mice, 41% and 89% of [^18^F]Favipiravir in the mouse brain and plasma remained at 30 min post-tracer administration, respectively, indicating moderate in vivo metabolic stability of [^18^F]Favipiravir.

Neurodegenerative diseases, including Alzheimer’s disease (AD), are known to disrupt brain integrity and/or the neuroimmune system, thus leading to an increased risk of SARS-CoV-2 infection, neuroinflammation, and immune compromise, as well as safety concerns for dose selection for antiviral therapy [25]. In this work, we also plan to investigate the neuro-pharmacokinetic profiles of [^18^F]Favipiravir in highly vulnerable brains impacted by neurodegeneration. Such critical information would substantially and rapidly improve our design of antiviral treatment plans, particularly for treating potential SARS-CoV-2 reservoirs in the CNS. In the experiments, we performed [^18^F]Favipiravir PET study in naive and transgenic mouse models of Alzheimer’s disease (5xFAD). In PET imaging studies with AD mice (Figure 2), [^18^F]Favipiravir demonstrated similar low brain penetration (0.52 SUV_peak_) and steady brain kinetics in AD and age-matched wild-type mice (Figure S1). There was no significant difference in the CNS distribution and retention of [^18^F]Favipiravir between AD and WT, which indicated that similar dosing could be utilized based on preclinical animal models. However, this cannot rule out the potential pharmacokinetic difference if the brain is under neurodegenerative conditions comorbid with SARS-CoV-2 infection. A further study using transgenic mouse models of AD with COVID-19 infection under a controlled laboratory setting of biological safety level 3 (BSL3) is necessary to validate this hypothesis. As a proof-of-concept and taking advantage of the translational nature of PET imaging in high species, we also evaluated [^18^F]Favipiravir in rhesus monkeys, which showed similar low brain penetration (0.61 SUV_peak_) and slow kinetics compared to rodents (Figure S2). Although the heterogeneous distribution of the radioactivity was observed for [^18^F]Favipiravir, the difference in the tracer uptake between various brain regions was marginal. This is somewhat expected for naive animals with intact BBB and low brain permeability of the radiolabeled drug. In addition, a higher uptake of [^18^F]Favipiravir was observed in the heart and lungs; the max uptake was 10 SUV in the heart and 4 SUV in the lung around 1 min post-tracer administration, followed by a rapid washout, and the SUV gradually declined to 0.4 (for the heart) and 0.2 (for lungs) over 60 min post-tracer administration (Figure S3).

## 4. Materials and Methods

### 4.1. Labeling of [^18^F]Favipiravir

Unless otherwise mentioned, all chemicals and solvents were purchased from Sigma-Aldrich (St. Louis, MO, USA). The target water containing [^18^F]fluoride (34 mCi) slowly passed through the Sep-Pak Accell Plus QMA Carbonate Plus Light Cartridge (Waters, cat. No. 186004540, Milford, MA, USA). The Sep-Pak Cartridge was eluted with a solution of base (e.g., K_2_CO_3_/K222, 2/10 mg) in acetonitrile and water (1 mL, *v*/*v* 7:3). The solution was heated to 110 °C with nitrogen gas passing through. When no liquid was left in the vial, anhydrous acetonitrile (1 mL) was added with nitrogen gas passing through. This step was repeated two more times. Then the vial was cooled with nitrogen gas passing through. A solution of Favipiravir precursor (methyl-5-chloroisoxazolo[4,5-b]pyrazine-3-carboxylate, 4 mg, San Diego, CA, USA) in DSMO (300 µL) was added to dried [^18^F]KF/K222, and the reaction was heated at 130 °C for 10 min. After that, the vial was cooled to 45 °C. Then NaOH (aq. 1 N, 0.4 mL) was added and heated at 110 °C for 15 min. After that, the vial was cooled to 45 °C, H_3_PO_4_ (aq., 0.2 M, 3 mL) was added, and the solution was ready for HPLC purification. A Phenomenex Luna C18 Prep Column (5 μm, 10 × 250 mm, Torrance, CA, USA) was used with an eluent of acetonitrile/H_2_O (*v*/*v* = 3/97, containing 0.01 M H_3_PO_4_) at 5 mL/min. The product was eluted at 16 min, and 6.7 mCi of [^18^F]Favipiravir was obtained in 29% decay-corrected RCY. The collected radioactivity was diluted with water (100 mL) and passed through the Oasis^®^ Plus HLB Cartridge (the efficiency for trapping tracer was about 60–70%, Waters, Milford, MA, USA). After the Oasis^®^ Plus HLB was washed with water (10 mL), [^18^F]Favipiravir was eluted with ethanol (1.5 mL). Then the solution was formulated in saline for PET imaging and biodistribution studies. The stability of the formulated [^18^F]Favipiravir was analyzed by HPLC radiochromatogram (a XBridge C18 column (5 µm, 4.6 mm × 100 mm, Waters, Milford, MA, USA) was used with an eluent of acetonitrile/H_2_O (*v*/*v* = 3/97, containing 0.01 M H_3_PO_4_) at 1 mL/min).

### 4.2. PET Imaging in Mice with [^18^F]Favipiravir

PET imaging was performed according to previous reports [26,27,28]. CD-1 mice (2- to 3-month-old, female), AD (5xFAD, female), and wild-type mice (10-month-old) were used in PET imaging with [^18^F]Favipiravir. The Genisys G4 PET/X-ray scanner (Sofie Biosciences, Dulles, VA, USA) was used with [^18^F]Favipiravir (ca. 0.04 mCi/100 µL), and the dynamic imaging was acquired for 1 h. For P-glycoprotein (P-gp) blocking experiments, a solution of Tariquidar (10 mg/kg) or Cyclosporin (25 mg/kg) in saline (300 µL) containing 10% ethanol and 5% Tween^®^ 80 was injected via the pre-embedded tail vein catheter 15 min before tracer injection. The data were analyzed with Amide and PMOD.

### 4.3. PET/MRI Imaging in NHP with [^18^F]Favipiravir

The PET imaging was performed according to the previous reports [26,28]. T1-weighted MR anatomical images were acquired on a 3.0 T scanner (GE Discovery 750, Milwaukee, WI, USA). The rhesus was anesthetized with ketamine (10 mg/kg; intramuscular injection). A circular 32-channel array head coil was placed on top of the monkey’s head. The whole-brain images were acquired with a 3D Bravo T1 sequence.

The male rhesus (weight range 9.48–10.96 kg) underwent PET (GE Discovery Elite 690, Chicago, IL, USA). The rhesus was anesthetized with ketamine (10 mg/kg; Intramuscular Injection), and then 2% isoflurane and 98% oxygen were used. A solution of [^18^F]Favipiravir (3.9–5.6 mCi) was injected, followed by a dynamic PET scan of the head or lung for 60 min.

### 4.4. Ex Vivo Whole-Body Biodistribution in Mice with [^18^F]Favipiravir

The biodistribution experiments were performed according to the previous reports [26,28]. At 5, 15, 30, and 60 min post-tracer administration, the organs of interest were collected and weighted, and the radioactivity in organs was measured by a gamma counter.

### 4.5. Radiometabolite Analysis with [^18^F]Favipiravir in Mice

The radiometabolite analysis was performed according to the previous reports [26,29]. The mouse brain and blood were collected post-[^18^F]Favipiravir administration. The mouse brain was homogenized and centrifuged, and the supernatant was analyzed using radio-HPLC. Both [^18^F]Favipiravir and ^18^F-metabolite were isolated by HPLC, and their radioactivities were measured by a gamma counter.

## 5. Conclusions

The PET evaluation of radiolabeled favipiravir ([^18^F]favipiravir) has provided valuable insights into the distribution, tissue uptake, pharmacokinetics properties, and clearance of the antiviral in naïve mice, transgenic mouse models of AD, and NHPs in vivo. The translational approach, using radiolabeling methods, could inform in vivo behavior of antiviral drugs and may be useful for evaluating other new or repurposed antivirals in the treatment of SARS-CoV-2 infection.

## Data Availability

Not applicable.

## Supplementary Materials

The following supporting information can be downloaded at: https://www.mdpi.com/1424-8247/16/4/524/s1, Table S1. Biodistribution of [^18^F]Favipiravir in mice (%ID/g); Figure S1. TACs of [^18^F]Favipiravir in AD mice and WT mice brain (*n* = 3); Figure S2. TACs of [^18F^]Favipiravir in NHP brain (*n* = 2); Figure S3. PET imaging of NHP with [^18F^]Favipiravir; Figure S4. Metabolic analysis of [^18^F]Favipiravir in mice brain and plasma at 30 min after [^18^F]Favipiravir administration (*n* = 2).
