# Supplementary material for: Evaluation of [18F]Favipiravir in Rodents and Nonhuman Primates (NHP) with Positron Emission Tomography
Source: Pharmaceuticals (Basel). 2023 Mar 31;16(4):524. doi: 10.3390/ph16040524 (PMC10146102; doi:10.3390/ph16040524)
Supplement: Supplementary file 1 [file pharmaceuticals-16-00524-s001.zip › pharmaceuticals-2240539-supplementary.pdf]

Table S1. Biodistribution of [^{18}F]Favipiravir in mice (%ID/g).

	5 min			15 min			30 min			60 min		
	**Mean**	**SD**	**N**	**Mean**	**SD**	**N**	**Mean**	**SD**	**N**	**Mean**	**SD**	**N**
Brain	2.33	0.07	4	2.71	0.18	4	2.67	0.10	4	1.84	0.17	4
Blood	9.53	0.27	4	8.14	0.54	4	7.27	0.37	4	4.83	0.23	4
Muscle	4.67	0.22	4	4.26	0.03	4	3.64	0.16	4	2.49	0.24	4
Spleen	5.23	0.19	4	4.54	0.18	4	3.84	0.37	4	2.66	0.14	4
Heart	5.55	0.33	4	4.82	0.26	4	3.89	0.40	4	2.57	0.25	4
Lung	7.85	0.32	4	6.92	0.49	4	6.28	0.40	4	3.86	0.37	4
Pancreas	4.85	0.21	4	4.59	0.37	4	4.14	0.17	4	2.93	0.23	4
Stomach	1.04	0.35	4	1.06	0.42	4	1.38	0.48	4	0.97	0.18	4
Small Intestine	5.61	0.75	4	5.29	0.62	4	5.46	0.20	4	4.30	1.02	4
Kidney	7.60	0.43	4	7.74	0.89	4	6.97	0.77	4	5.18	0.47	4
Liver	7.46	0.61	4	6.66	0.44	4	6.27	0.39	4	4.43	0.61	4
Bone	6.10	0.97	4	6.27	1.24	4	6.00	0.68	4	6.05	1.22	4

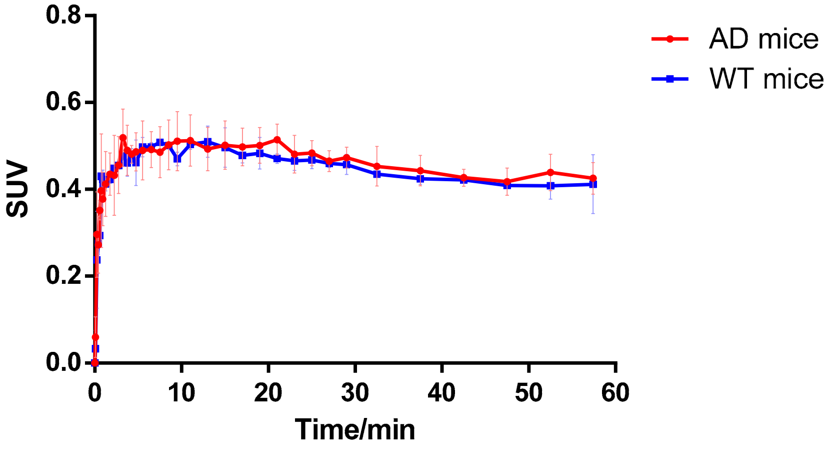

Figure S1. TACs of [^{18}F]Favipiravir in AD mice and WT mouse brains (n = 3).

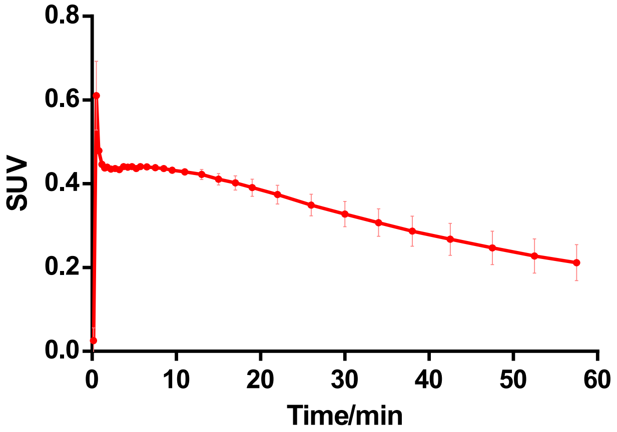

Figure S2. TACs of [^{18}F]Favipiravir in NHP brains (n = 2).

a

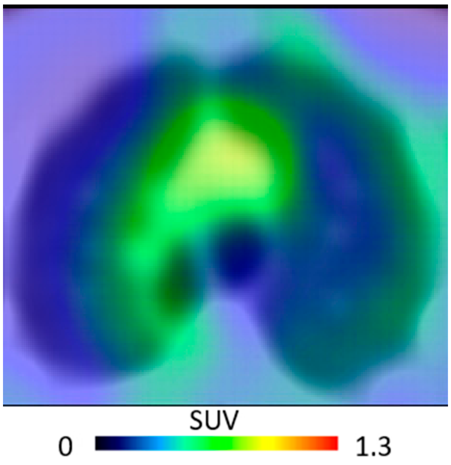

b.

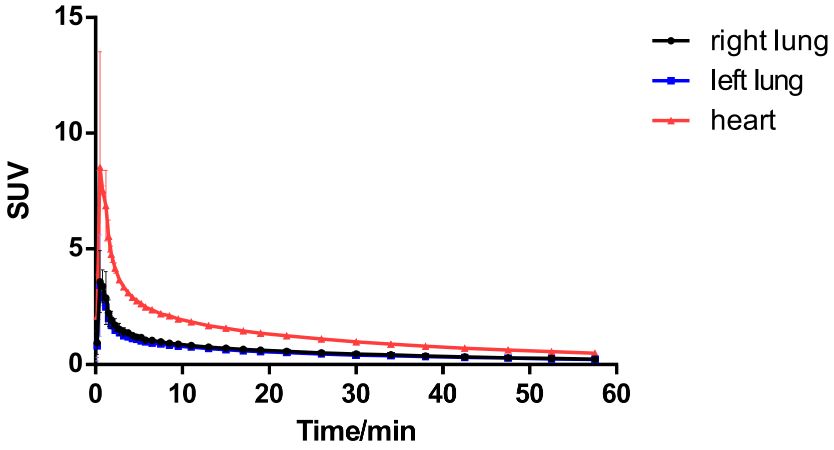

c.

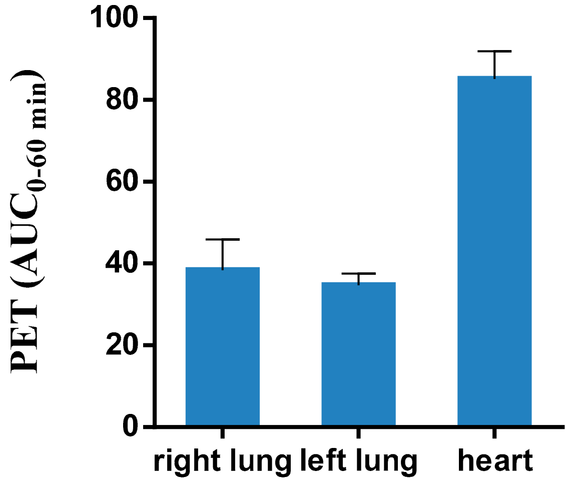

Figure S3. PET imaging of NHP with [^{18}F]Favipiravir. (a) Representative summed PET image (0-60 min) of NHP lungs and heart with [^{18}F]Favipiravir (*n* = 2); (b) TACs (0-60 min) of [^{18}F]Favipiravir in the NHP lungs and heart (*n* = 2); (c) AUCs (0-60 min) of [^{18}F]Favipiravir in the NHP lungs and heart (*n* = 2).

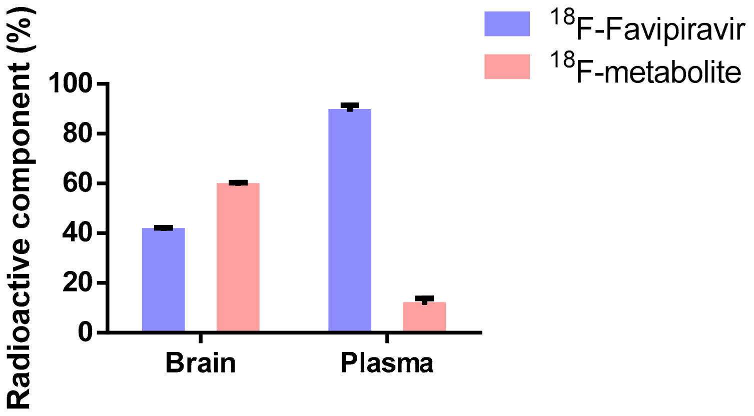

Figure S4. Metabolic analysis of [^{18}F]Favipiravir in mouse brain and plasma at 30 min after [^{18}F]Favipiravir administration (*n* = 2).
